# Soil bacterial diversity and community structure of *Suaeda glauca* vegetation in the Hetao Irrigation District, Inner Mongolia, China

**DOI:** 10.3389/fmicb.2024.1358783

**Published:** 2024-06-13

**Authors:** Ruixiao Dong, Xinbo Wang, Yuyi Li, Hongyuan Zhang, Xiaobin Li, Jiashen Song, Fangdi Chang, Wenhao Feng, Huancheng Pang, Jing Wang

**Affiliations:** State Key Laboratory of Efficient Utilization of Arid and Semi-arid Arable Land in Northern China (the Institute of Agricultural Resources and Regional Planning, Chinese Academy of Agricultural Sciences), Beijing, China

**Keywords:** *Suaeda glauca*, saline-alkali soil, rhizosphere, bacteria, community structure

## Abstract

Exploring the bacterial community in the *S. glauca* rhizosphere was of great value for understanding how this species adapted to the saline-alkali environment and for the rational development and use of saline-alkali soils. In this study, high-throughput sequencing technology was used to investigate the diversity characteristics and distribution patterns of soil bacterial communities in the rhizosphere of *S.glauca*-dominated communities in the Hetao Irrigation Distract, Inner Mongolia, China. The relationships among bacterial characteristics, soil physicochemical properties and vegetation in four sampling sites were analyzed. The soil bacterial communities in the rhizosphere of *S. glauca*-dominated communities were mainly composed of 16 phyla (i.e., *Proteobacteria, Actinobacteria, Bacteroidetes, Gemmatimonadetes, Chloroflexi, Acidobacteria, Firmicutes, Planctomycetes, Deinococcus-Thermus, Verrucomicrobia, Saccharibacteria, Cyanobacteria, Nitrospirae, JL-ETNP-Z39, Parcubacteria* and *Chlorobi*), and these populations accounted for more than 99% of the total bacterial community. At the genus level, the main bacterial communities comprised *Halomonas, Nitriliruptor, Euzebya* and *Pelagibius*, which accounted for 15.70% of the total bacterial community. An alpha diversity analysis indicated that the richness and diversity of rhizosphere soil bacteria differed significantly among the sampling sites, and the bacterial richness and diversity indices of severe saline-alkali land were higher than those of light and moderate saline-alkali land. The principal component analysis (PCA) and linear discriminant analysis effect size (LEfSe) showed significant differences in the species composition of the rhizosphere soil bacterial community among different sampling sites. A correlation analysis showed that the number of bacterial species exhibited the highest correlation with the soil water content (SWC). The richness and evenness indices were significantly correlated with the SWC and SO_4_^2–^, K^+^ and Mg^2+^ concentrations. The electrical conductivity (EC), soluble ions (Na^+^, CO_3_^2–^ + HCO_3_^–^, K^+^, Ca^2+^, Mg^2+^, and SO_4_^2+^), SWC and vegetation coverage (VC) were the main drivers affecting the changes in its community structure. The bacterial community in the rhizosphere of *S. glauca* enhanced the adaptability of *S. glauca* to saline-alkali environment by participating in the cycling process of nutrient elements, the decomposition of organic matter and the production of plant growth regulating substances. These results provided a theoretical reference for further study on the relationship among rhizosphere soil microorganisms and salt tolerance in halophytes.

## 1 Introduction

Saline-alkali land is widely distributed in Hetao Irrigation District of Inner Mongolia, and the area affected by saline-alkali is as high as 4.3 × 10^5^ hm^2^. The types of saline-alkali land are diverse and the salt composition is complex. Sulfate salinized soil, chloride salinized soil, soda salinized soil and alkalized soil coexist in combination, with varying levels of salinity and alkalinity, which is highly typical and representative (Zhang et al., 2018). In the saline-alkali land of Hetao Irrigation District, saline-alkali stress destroys soil structure, induces soil particle dispersion, causes soil compaction, poor ventilation and permeability, reduces fertility, and inhibits the growth and activity of microorganisms, thus deteriorating soil physical, chemical and biological properties. In addition, saline-alkali stress can induce osmotic stress and specific ion toxicity, hinder the absorption and utilization of water and nutrients by plants, affect the normal growth of crops, and lead to slow growth or even death of crops (Oumi et al., 2014; Fahad et al., 2015). Therefore, saline-alkali stress is an important factor that restricts plant growth and agricultural development and harms the ecological environment in the region (Cui et al., 2009). At present, phytoremediation has become a strategy to alleviate saline-alkali stress, which both provides ecological benefits and increases crop productivity, and these saline-alkali land improvements related to phytoremediation are stable and durable. Halophyte communities, including *S. glauca*, *Phragmites australis*, *Tamarix chinensis* and *Sophora alopecuroides*, are a valuable resource in saline-alkali ecosystems (Edgar, 2013). These salt-tolerant plants can be cultivated for the adaptive improvement of saline-alkali land (Jesus et al., 2015).

Bacteria play critical roles in plant growth, development and adaptation to environmental changes (Zhou et al., 2016). Bacteria can form a variety of symbiotic systems with vegetation for adaptation to complex habitats. In a saline-alkali environment, bacteria participate in multiple physiological and ecological processes of the vegetation and regulate its functional traits (e.g., promoting efficient nutrient and water uptake and improving stress resistance) (Waller et al., 2005; Mucciarelli et al., 2010). Bacteria produce large amounts of organic acids during their life activities by continuously and slowly releasing available forms of nitrogen, phosphorus and potassium into the soil. This process can effectively modify the physical, chemical and biological properties of soil, improve soil fertility in saline-alkali land, and play a role in improving saline-alkali land through vegetation restoration. The rhizosphere is the site of the most direct interactions among plants, soil and microorganisms and exhibits the highest microorganism activity in plants. Therefore, understanding the diversity and community structure of rhizosphere soil bacteria can provide important information on the adaptation of plants to the saline-alkali environment and even improve this process (Raiesi and Salek-Gilani, 2018). However, the current research on rhizosphere bacteria in saline-alkali land is mostly limited to the determination and analysis of pure culture method, and some bacterial species are difficult to be successfully cultured. In addition, the research of rhizosphere bacteria in saline-alkali land often only involves coastal saline-alkali land, and does not take into account the differences in bacterial groups among different types of saline-alkali land (Sun et al., 2020), which limits our comprehensive understanding of the functions of rhizosphere soil bacteria and their effects in regulating vegetation growth and stress adaptation in saline-alkali wasteland.

*S. glauca* is a typical indicator plant of saline-alkali land (Kong and Zheng, 2015). This species is a common saline-alkali-tolerant plant in the Hetao Irrigation District and an important pioneer plant in the improvement of saline-alkali land. The extremeness, uniqueness and diversity of its habitats may drive the microbial communities inhabiting these habitats to yield a rich diversity of species through long-term adaptation. To date, few studies have investigated *S. glauca* and the composition and driving factors of soil bacterial communities in its habitats. A previous study found that the soil bacterial communities in the rhizosphere of *Chenopodiaceae* halophytes are dominated by *Actinobacteria* and *Proteobacteria* and exhibit a rich diversity, that the soil bacterial communities in the rhizosphere of *Zygophyllaceae* halophytes are dominated by *Proteobacteria*, and that *Tenericutes* comprises the main abundant phyla of *Tamaricaceae* halophytes (Zhao S. et al., 2016). In the same salinized habitat, Cl^–^ in the rhizosphere soil is one of the main drivers affecting the variation in the soil bacterial communities in the rhizosphere of different halophytes (Zhao Y. et al., 2016). Moreover, a previous study found that *Proteobacteria* and *Firmicutes* were the dominant bacteria in the rhizosphere soil of Chenopodiaceae plants such as *Kalidium foliatum* and that the bacterial diversity was positively correlated with the levels of soil organic carbon, organic matter and total nitrogen and negatively correlated with the soil pH and EC (Li et al., 2018).

In the present study, four typical saline-alkali wasteland sites with *S. glauca* as the constructive species (> 95% of the total vegetation) was selected. They can reflect different degrees of saline-alkali stress and are representative in terms of land type, which can comprehensively assess the dynamics of rhizosphere bacterial communities in this area. We analyzed the bacterial communities in rhizosphere soils of *S. glauca* collected from the four sampling sites with the aim of exploring (1) whether there are significant differences in the diversity of rhizosphere bacterial communities of *S. glauca* at different sampling sites? (2) What are the factors affecting the bacterial community structure in the rhizosphere of *S. glauca*? (3) Can the rhizosphere bacterial community improve the adaptability of *S. glauca* to saline-alkali environment?

We hypothesized that there were significant differences in the diversity of rhizosphere bacterial communities of *S. glauca* at different sampling sites, and the diversity of rhizosphere bacterial communities under high saline-alkali stress was significantly lower than that under mild saline-alkali stress. There were significant differences in the rhizosphere bacterial community structure of *S. glauca* at different sampling sites, and soil indicators, especially salt content and pH value, were the main factors contributing to the variation in rhizosphere bacterial community structure. By understanding the composition of rhizosphere bacterial communities at different sampling sites, it was found that the main functional bacteria of each sampling sites could improve the adaptability of *S. glauca* to saline-alkali environment.

## 2 Materials and methods

### 2.1 Study area

This study was conducted in the Hetao Irrigation District (40°12′–41°21′N and 106°10′–109°30′E), which is one of the greatest large-scale irrigation districts of the Yellow River in China. The administrative division of this area included most parts of Bayannur City (e.g., Wuyuan and Dengkou County, Linhe District, and Urad Front and Rear Banner) and small parts of Baotou City in Inner Mongolia, covering an area of approximately 11,600 km^2^. The terrain in the area was relatively flat, and the soil quality was better than that in the surrounding area. The topography was slightly inclined from the southwest to the northeast, and the altitude ranged from 1020 to 1050 m. The area had a continental climate with a large temperature difference between day and night. The average annual temperature was 7.6°C, and the average annual precipitation was approximately 159.8 mm (Du et al., 2024).

### 2.2 Plant and soil sampling

**FIGURE 1:**
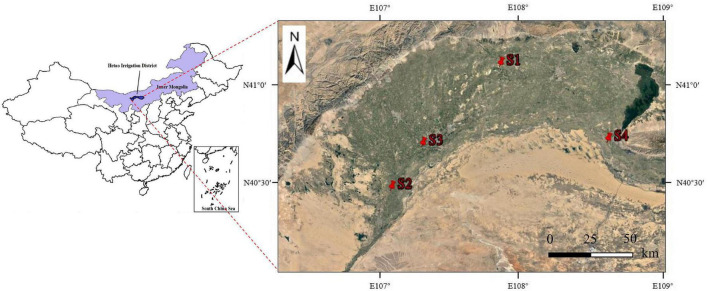
Location of the Hetao Irrigation District, Inner Mongolia, China, and distribution of the sampling sites.

In this study, four sampling sites showing typical distributions of *S. glauca*-dominated communities (*S. glauca* accounted for >95% of the total vegetation) were selected in the Hetao Irrigation District. These sites generally covered the typical distribution areas of *S. glauca* vegetation in the Hetao Irrigation District and included desert grassland(S1), waterfront wild grassland(S2), wild grassland near farmland(S3) and wild grassland near grazing land(S4). The selected sites were extracted using global positioning system (GPS) coordinates. The distribution of the sites was shown in Figure 1.

The vegetation coverage (VC) of *S. glauca* was determined by the percentage of the vertical projection area of vegetation on the ground to the sample area. In addition, the aboveground parts of plants in the sample plot were collected and dried to record the constant weight and thus obtain the above-ground biomass.

Soil samples were collected at a depth of 0–15 cm in August 2016. Plant residues were removed before sampling. From each site, five soil samples were mixed into a composite sample. Another five soil samples were collected 5 m away and mixed to obtain another composite sample. A total of three composite samples were collected from each site. Parts of the field-moist samples were maintained in craft paper bags on ice packs (for 2–6 h) and then stored at −80°C for the determination of soil microbial DNA. The subsamples were sealed in Ziploc bags to analyze their soil water content (SWC), pH, EC and water-soluble and exchangeable ions.

### 2.3 Measurement of soil physical and chemical properties

All the soil samples stored in sealed Ziploc bags were oven-dried to determine their SWC and then finely ground to pass through a 2-mm sieve for the analysis of soil chemical properties. The water-soluble ions in the soil were extracted using a 1:5 soil: water suspension that was stirred for 5 min using a digital oscillator (Changzhou Runhua Electric Appliance Co., Ltd., China) and filtered using filter paper. The levels of pH and EC of the saturated extract were then determined using digital pH and EC meters (Mettler Toledo International Trade (Shanghai) Co., Ltd., China), respectively. The concentrations of water-soluble ions (e.g., K^+^, Na^+^, Ca^2+^, Mg^2+^, Cl^–^, SO_4_^2–^, CO_3_^2–^ and HCO_3_^–^) were then determined using the method described by Zhao et al. (2018).

### 2.4 DNA extraction, PCR amplification, Illumina MiSeq sequencing and sequencing data processing

Soil genomic DNA from four soil samples was extracted using the E.Z.N.A. Mag-Bind Soil DNA Kit (Omega Bio-tek, Norcross, GA, USA) following the manual (Liu et al., 2020). The extracted DNA were then confirmed by 1% agarose gel electrophoresis and stored at −20°C for further analysis (Liu et al., 2021). The specific bar-coded primer pair 515F (5′-GTGCCAGCMGCCGCGG-3′) and 907R (5′-CCGTCAATTCMTTTRAGTTT-3′) was used to amplify the V3-V4 region of the bacterial 16S rRNA gene. The 515F and 907R primer pairs were designed to bind to the 16S rRNA genes of most bacterial species, so they had good versatility and could be used for a wide range of bacterial community studies (Biddle et al., 2008). For each soil sample, a 10-digit barcode sequence was added to the 5′ end of the forward and reverse primers. The PCR amplification was conducted in a 20-mL reaction system using TransGen AP221-02 and an ABI GeneAmp^®^ 9700 (ABI, Carlsbad, USA) under the following conditions: initial denaturation at 95°C for 5 min followed by 25 cycles of 95°C for 30 s, 55°C for 30 s and 72°C for 30 s and a final extension at 72°C for 10 min. The PCR reactions were performed in triplicate 25-mL mixtures containing 2.5 mL of 10 × Pyrobest Buffer, 2 mL of 2.5 mM dNTPs, 1 mL of each primer (10 mM), 0.4 U of Pyrobest DNA Polymerase (TaKaRa), and 15 ng of template DNA. The amplicon mixture was used for analysis with a MiSeq Genome Sequencer (Illumina). The PCR products were extracted from 2% agarose gels and purified using an AxyPrep DNA Gel Extraction Kit (Axygen, Union City, CA, USA) (Liu et al., 2020). Thereafter, the products were quantified using QuantiFluor™-ST (Promega, Madison, USA) and then mixed at appropriate ratios according to the sequencing size requirement equally before pyrosequencing. The purified amplicons were pooled in equimolar amounts and paired-end sequenced (2 × 300) on an Illumina MiSeq platform (Allwegene, Beijing) using standard protocols (Jing et al., 2018).

The obtained original sequences were spliced and filtered using Flash v1.2.11 and Trimmomatic v0.33 software to obtain high-quality sequences. The UCLUST consensus taxonomy assigner (UCLUST v1.2.22q) was used to cluster the sequences with 97 % or higher similarity into operational classification units (OTUs) (Edgar, 2010). Based on the Silva database containing bacterial and archaeal 16S ribosomal RNA genes (Release 119), the RDP Classifier (70% confidence threshold) was utilized to comparatively analyze the representative sequences from the OTUs and then annotate the species information for the community at various levels (kingdom, phylum, class, order, family, genus and species) (Liu et al., 2021). The software and algorithms used in the analyses are included in the QIIME platform.

### 2.5 Data analysis

According to the results of the taxonomic analysis, we used the graphing tool in R (v3.6.0) to plot the bar plots showing the species composition at each taxonomic level (Oberauner et al., 2013). Based on an Unweighted UniFrac distance matrix, we clustered the samples and constructed trees based on the unweighted pair group method with arithmetic mean (UPGMA). QIIME (version 1.8) analysis software was used to analyze the alpha diversity of the samples and calculate the Chao1 and Shannon indices at a similarity of 0.97, and R (v3.6.0) software was used to plot the results (Schloss et al., 2011). R (v3.6.0) software was used to draw the diagram of the PCA results based on the OTU information (Mitter et al., 2017). Based on taxonomic composition, linear discriminant analysis (LDA) effect size (LEfSe) analysis was performed to identify species with significant differences in abundance between the rhizosphere soils of *S. glauca* at different sampling sites and to construct cladograms (Segata et al., 2011).

Statistical tests were performed using SPSS software (v. 19.0, SPSS Inc., Chicago, IL, USA). When the ANOVA generated a significant *F*-value (*P* < 0.05) for the treatments, the treatment means were compared using the LSD test. In addition, we used redundancy analysis (RDA) techniques to examine the relationships between environmental variables and bacterial communities. RDA was performed using CANOCO 4.5 (Microcomputer Power, Ithaca, NY, USA).

## 3 Results

### 3.1 Analysis of the vegetation status and soil physicochemical properties at different sites

Specific information of the sampling sites is shown in Table 1. All four sites were flat, and the soil type was silty loam. However, the land type was slightly different. S1 was desert grassland; S2 was waterfront wild grassland; S3 was wild grassland near farmland; and S4 was wild grassland near grazing land. Despite *S. glauca* vegetation was observed in all the sites, the vegetation coverage (VC), number of *S. glauca* (NS), height of *S. glauca* (HS) and above-ground biomass (AGB) differed significantly. Among all plots, the HSand AGB were highest in the S4 plots and lowest in the S2 plots. The highest VC was observed in the S2 plots, and the lowest was detected in the S3 plots. The highest NS was found in the S1 plots, and the lowest was detected in the S4 plots. No obvious regularity in the differences among the sampling sites with different vegetation factors was observed. Differences in vegetation characteristics among sampling sites inevitably affect the composition of microbial communities.

**TABLE 1:** Information on the sampling sites.

Sampling site	Location	Geographic coordinates	Altitude (m)	Soil texture	Vegetation coverage (%)	Number of *Suaeda glauca* (m^2^)	Height of *Suaeda glauca* (cm)	Above- ground biomass (g ⋅ m^–2^)
S1	Hudeng Gebu village, Wuyuan County	41° 7′45.45′′N, 107°51′22.21”E	1015	Silt loam	55.0	86.7	11.9	730.7
S2	Xinhe village, Dengkou County	40°29′25.50′′ N, 107° 4′55.12′′ E	1038	Silt loam	61.0	72.7	5.2	543.7
S3	Houjia Gedang village, Linhe District	40°43′5.94′′ N, 107°18′13.60′′ E	1021	Silt loam	44.7	84.3	10.0	871.7
S4	Tabu village, Urad Front Banner	40°42′54.61′′ N, 108°35′10.92′′ E	1002	Silt loam	50.7	64.3	20.0	926.5

The land type was slightly different. S1 was desert grassland; S2 was waterfront wild grassland; S3 was wild grassland near farmland; and S4 was wild grassland near grazing land.

As shown in Table 2, the soil in the different sampling sites was alkaline. The pH was always greater than 9 in all the sites and even exceeded 10 in the S1 plots. The EC also significantly differed among different sampling sites (*p* < 0.05), and the EC was highest in the S3 plots, followed by the S1 plots and then the S4 plots and finally the S2 plots: S3 was severe saline-alkali land, S1 was moderate saline-alkali land, and S4 and S2 were light saline-alkali land. Based on the classifications of the saline-alkali types, S3 was a soda saline-alkali soil, S1 and S4 were soda-chloride saline soils, and S2 was a soda-sulfate-chloride saline soil. The SWC was significantly different among the different sampling sites (*P* < 0.05), and the SWC was highest in the S2 plots, followed by the S1 plots, then in the S4 plots, and finally in the S3 plots; the highest and lowest levels were 23.8 and 17.2%, respectively.

**TABLE 2:** Physical and chemical properties of soil samples at each site.

Sampling site	pH	EC (μS ⋅ cm^–1^)	Soluble ions (cmol ⋅ kg^–1^)							SWC (%)
			K^+^	Na^+^	Ca^2+^	Mg^2+^	SO_4_^2–^	Cl^–^	HCO_3_^–^+CO_3_^2–^	
S1	10.0 ± 0.03a	984 ± 5.00b	0.62 ± 0.00a	3.85 ± 0.04b	0.42 ± 0.00a	0.46 ± 0.00a	2.23 ± 0.01a	3.84 ± 0.02a	4.33 ± 0.02b	21.80 ± 0.08b
S2	9.4 ± 0.05c	315 ± 1.61d	0.08 ± 0.00d	1.07 ± 0.01d	0.09 ± 0.00d	0.08 ± 0.00c	1.13 ± 0.01b	1.01 ± 0.01d	1.59 ± 0.01d	23.80 ± 0.15a
S3	9.8 ± 0.05b	2463 ± 12.25a	0.13 ± 0.00b	8.12 ± 0.04a	0.18 ± 0.00b	0.04 ± 0.00d	0.58 ± 0.00d	2.47 ± 0.01c	27.88 ± 0.14a	17.20 ± 0.08d
S4	9.2 ± 0.04d	454 ± 2.31c	0.09 ± 0.00c	1.32 ± 0.01c	0.16 ± 0.00c	0.16 ± 0.00b	0.80 ± 0.00c	2.54 ± 0.01b	2.19 ± 0.01c	19.90 ± 0.08c

The physical properties include the SWC, (i.e., soil water content). The chemical properties include the EC, (i.e., electrical conductivity), pH and soluble ions. The different letters in the same column indicate a significant difference among groups by one-way ANOVA (Duncan, *p* < 0.05).

### 3.2 Diversity analysis of rhizosphere bacterial communities at different sites

#### 3.2.1 Alpha diversity of rhizosphere bacterial communities at different sites

**FIGURE 2:**
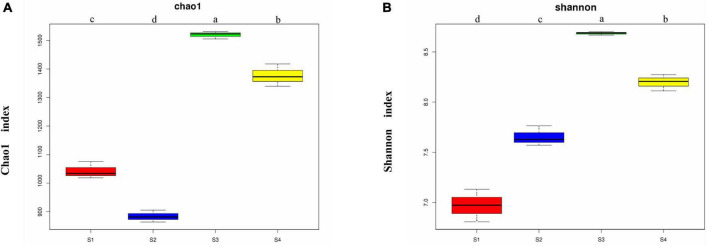
Chao1 indexes **(A)** and Shannon indexes **(B)** of the bacterial communities from the rhizosphere of *Suaeda glauca* at different sampling sites. S1, desert grassland; S2, waterfront wild grassland; S3, wild grassland near farmland; and S4, wild grassland near grazing land. The horizontal bars within boxes represent medians. The tops and bottoms of boxes represent the 75th and 25th percentiles, respectively. The upper and lower whiskers extend to data no more than 1.5 × the interquartile range from the upper and lower edges of the box, respectively.

The Chao1 index obtained in the α-diversity analysis (Figure 2) showed that the soil bacterial richness levels of the four sampling sites were significantly different (*p* < 0.05). The richness of the bacterial community was highest in the S3 plots, followed by the S4 plots, the richness of both of these plots were markedly higher than that of the S1 and S2 plots, and the lowest richness was detected in the S2 plots. The Shannon index showed significant differences in the bacterial community diversity among different sampling sites (*p* < 0.05), and the community diversity was highest in the S3 plots followed by the S4 plots, then the S2 plots, and finally the S1 plots. The S3 plots with the highest salt content exhibited the highest bacterial community richness and diversity.

#### 3.2.2 Beta diversity of rhizosphere bacterial communities at different sites

**FIGURE 3:**
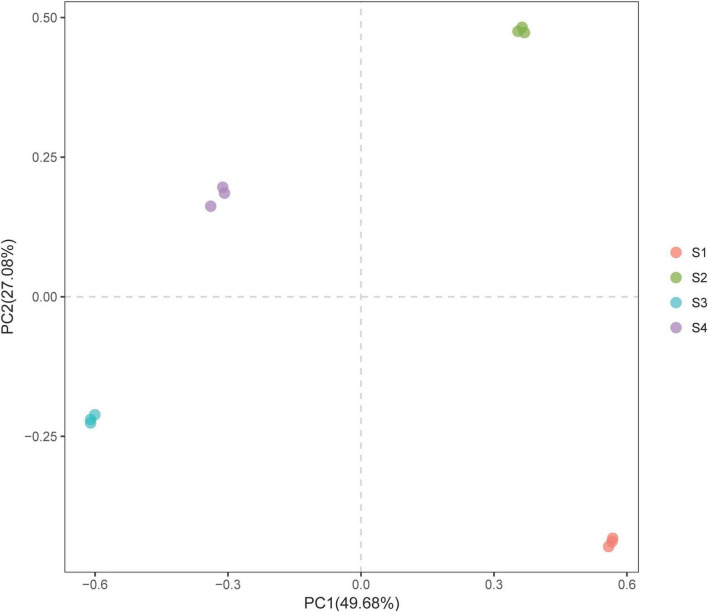
PCA plot of the bacterial communities in the rhizosphere soils of *S. glauca* at different sampling sites.

We conducted a PCA of the bacterial community composition under the different treatments (i.e., while considering the sequence abundance of OTUs). The results showed that although *S. glauca* vegetation was detected in all the sites, the structure or characteristics of the bacterial community under the different treatments varied due to differences in soil conditions (Figure 3). This variation led to differentiation on the PC axis, which clearly distinguished the bacterial communities among the different treatments.

### 3.3 Analysis of the structure of the rhizosphere bacterial communities at different sites

#### 3.3.1 Structure analysis of rhizosphere bacterial communities at different sites

**FIGURE 4:**
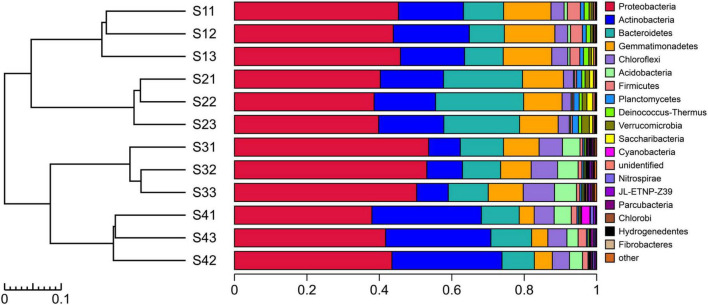
UPGMA cluster tree combined with a histogram of the phyla found in the rhizosphere soils of *S. glauca* at different sampling sites.

The experimental results showed that more than 99.67% of the species in the bacterial communities subjected to the different treatments could be classified to the phylum level (Figure 4). The top five dominant bacterial phyla in all the different treatments were *Proteobacteria, Actinobacteria, Gemmatimonadetes, Bacteroidetes* and *Chloroflexi*. Notably, *Proteobacteria* was the most abundant phylum in all the treatments, with relative abundances of 39.55–52.34%, but the relative abundance of dominant species differed among the treatments. The relative abundance of *Actinobacteria* decreased in the S3 plots compared with other treatments, and the bacterial taxa of *Proteobacteria*, *Acidobacteria* and *Chloroflexi* exhibited the opposite pattern. The relative abundance of *Bacteroidetes* was higher in the S2 plots than in the other treatments. Compared with the other treatments, *Gemmatimonadetes* and *Firmicutes* were found at a greater relative abundance in the S1 treatment. In addition to the abovementioned dominant phyla, the species with high abundance at the phylum level mainly included *Acidobacteria*, *Firmicutes*, *Planctomycetes*, *Deinococcus-Thermus*, *Verrucomicrobia*, *Saccharibacteria*, *Cyanobacteria*, *Nitrospirae*, *JL-ETNP-Z39*, *Parcubacteria* and *Chlorobi*, and these 16 populations accounted for 99.31% of the total bacterial communities.

**FIGURE 5:**
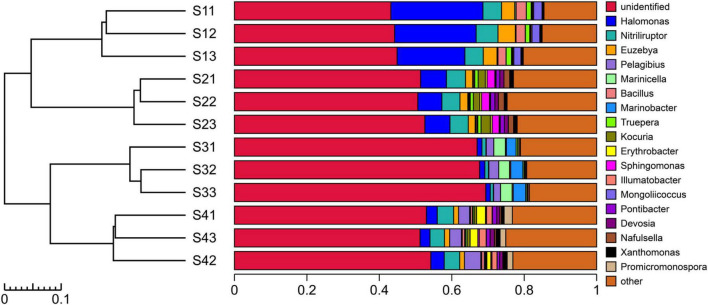
UPGMA cluster tree combined with a histogram of the genera in the rhizosphere soils of *S. glauca* at different sampling sites.

As shown in Figure 5, the experimental results showed that only 45.88% of the species in the bacterial communities of the different treatment plots could be classified to the genus level. Different sampling site treatments altered the composition of dominant bacteria at the genus level. The top five bacterial genera in the S1 plots were mainly *Halomonas*, *Nitriliruptor*, *Euzebya*, *Bacillus* and *Mongoliicoccus*, whereas those in the S2 plots were *Halomonas*, *Nitriliruptor*, *Kocuria*, *Sphingomonas* and *Euzebya*; in addition, those in the S3 plots were *Marinobacter*, *Marinicella*, *Pelagibius*, *Methylohalomonas* and *Halomonas*, and those in the S4 plots were *Nitriliruptor*, *Pelagibius*, *Halomonas*, *Erythrobacter* and *Promicromonospora*. Overall, the dominant species at the genus level (relative abundance > 1%) mainly comprised *Halomonas*, *Nitriliruptor*, *Euzebya* and *Pelagibius*, which accounted for 15.70% of the total bacterial community. In addition, *Halomonas* is a bacterial genus distributed in all the treated plots and plays an important role in the adaptation and resistance to saline-alkali stress.

#### 3.3.2 Linear discriminant analysis effect size (LEfSe) of the species composition at different sites

**FIGURE 6:**
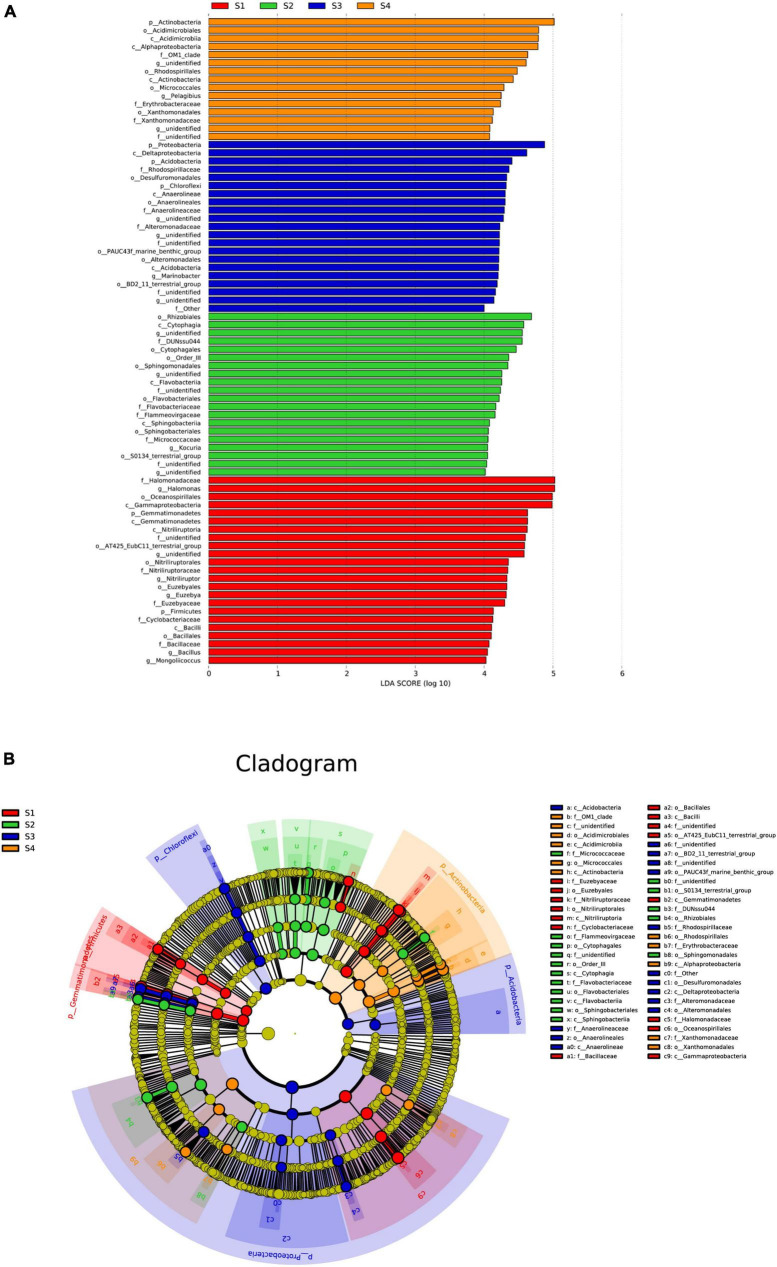
Effect size (LEfSe) analysis of the bacterial abundance **(A)** and cladograms (taxonomic trees) of bacteria **(B)** in the rhizosphere soils of *S. glauca* at different sampling sites. Linear discriminant analysis (LDA) scores > 4 identified bacterial biomarkers.

The abovementioned analysis could reveal that the microbial community composition showed significant differences among the different treatments; thus, an LEfSe analysis was used to identify species with significant differences in abundance among the treatments, and only those with LDA scores greater than 4 are shown (Figure 6). In the S1 plots, the main bacterial markers at the phylum level were *Gemmatimonadetes* and *Firmicutes*. In addition, in the S3 plots, the main bacterial markers were *Proteobacteria*, *Acidobacteria* and *Chloroflexi*, and in the S4 plots, the main bacterial marker included *Actinobacteria.*

At the genus level, the main bacterial markers in the S1 plots were *Halomonas, Nitriliruptor, Euzebya*, *Bacillus* and *Mongoliicoccus*, where those in the S2, S3 and S4 plots included *Kocuria*, *Marinobacter*, and *Pelagibius*, respectively.

### 3.4 Relationship between rhizosphere bacterial communities and environmental factors at different sites

**FIGURE 7:**
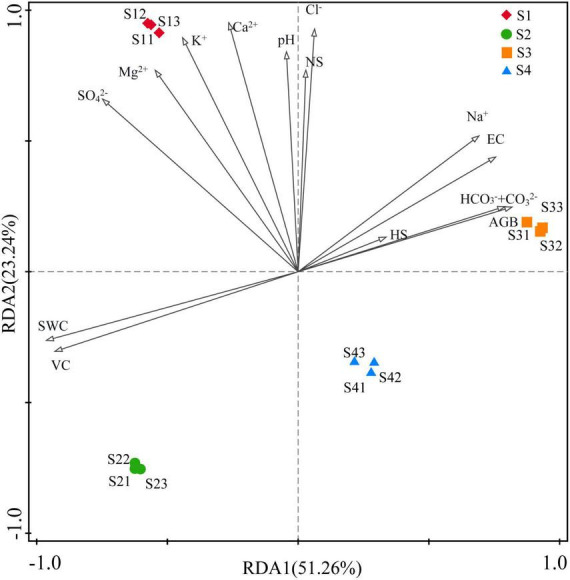
Redundancy analysis (RDA) between environmental factors and bacterial communities. (S1, S2, S3, and S4 represent the bacterial communities in the rhizosphere soils at four sampling sites, and environmental factors are represented by black lines with arrows).

The RDA indicated that environmental factors exerted strong influences on the rhizosphere bacterial community and explained 51.26% and 23.24% of the community variances between samples, respectively. However, the main influencing factors of rhizosphere bacterial community were different in different treatments. As shown in Figure 7, the bacterial community was mainly affected by K^+^, Ca^2+^ SO_4_^2–^and Mg^2+^ in the S1 treatment; by VC and SWC in the S2 treatment; by HS, AGB, HCO_3_^–^+CO_3_^2–^, pH, Na^+^ and EC in the S3 treatment; and by Mg^2+^, SO_4_^2–^, K^+^ and Ca^2+^ in the S4 treatment.

The physicochemical properties of soil had an important influence on the growth and development of plants in saline-alkali land. The correlation between the aboveground flora and soil physical and chemical indexes in Hetao Irrigation District was analyzed. The results from the correlation analysis between the ground flora and soil physicochemical indicators in areas of *S. glauca* in the Hetao Irrigation District showed that the number of *S. glauca* was mainly determined by the soil pH, as revealed by an extremely significant positive correlation between these variables (Table 3). The plant height was not significantly correlated with soil indicators. The VC was extremely significantly positively correlated with the SWC and negatively correlated with soil EC and HCO_3_^–^+CO_3_^2–^ concentration, and the AGB was significantly negatively correlated with the SWC. These results indicate that the growth of *S. glauca* was under dual control by soil water and salt and closely related with the soil alkalinity in the saline-alkali land of the Hetao Irrigation District.

**TABLE 3:** Relationships between environmental variables and bacterial communities.

	SWC	pH	EC	K^+^	Na^+^	Ca^2+^	Mg^2+^	SO_4_^2–^	Cl^–^	HCO_3_^–^+CO_3_^2–^	NS	HS	VC	AGB
NS	-0.2096	0.9816**	0.6844	0.6206	0.7516*	0.5944	0.3433	0.4269	0.4540	0.5657				
HS	-0.4120	-0.3721	-0.1332	0.0287	-0.1568	0.1967	0.2210	-0.1065	0.4987	-0.1706				
VC	0.9979**	-0.1144	-0.7803*	0.2001	-0.7368	0.0107	0.3114	0.5562	-0.3252	-0.7911 *				
AGB	-0.8556*	-0.1035	0.4343	-0.0901	0.3961	0.1197	-0.0621	-0.3925	0.4912	0.4168				
CHAO	-0.9762**	-0.0392	0.6815	-0.3007	0.6268	-0.1051	-0.3697	-0.6303	0.2568	0.7112	-0.0061	0.5454	-0.9878**	0.9161**
Shannon	-0.7613 *	-0.3433	0.5414	-0.7685*	0.4517	-0.6426	-0.8399*	-0.9548**	-0.3475	0.6790	-0.2191	0.1816	-0.7711 *	0.5742
*p__Acidobacteria*	-0.9646**	-0.0204	0.7638 *	-0.4264	0.7013	-0.2559	-0.5443	-0.7401	0.0726	0.8243 *	0.0581	0.3328	-0.9664 **	0.7877 *
*p__Actinobacteria*	-0.1167	-0.7445	-0.4726	-0.2797	-0.5196	-0.1646	-0.0069	-0.2541	0.0890	-0.4390	-0.8351*	0.8880 *	-0.1805	0.5758
*p__Bacteroidetes*	0.0965	-0.3724	0.0545	-0.8069 *	-0.0100	-0.8877*	-0.8827 *	-0.6698	-0.9423**	0.2352	-0.1949	-0.6136	0.1083	-0.4112
*p__Chloroflexi*	-0.9748 **	0.1060	0.8409 *	-0.3437	0.7879 *	-0.1802	-0.4967	-0.6764	0.1237	0.8862 *	0.1869	0.2579	-0.9687 **	0.7518 *
*p__Firmicutes*	-0.2328	0.2849	0.0002	0.7008	0.0500	0.8134 *	0.7864 *	0.5287	0.9354 **	-0.1643	0.1134	0.7300	-0.2509	0.5617
*p__Gemmatimonadetes*	-0.3342	0.8852 *	0.8091 *	0.3647	0.8513 *	0.3464	0.0540	0.1557	0.2451	0.7422	0.9562 **	-0.5770	-0.2726	-0.1184
*p__Proteobacteria*	-0.9663**	0.1706	0.8793 *	-0.3133	0.8307 *	-0.1585	-0.4880	-0.6495	0.1252	0.9202 **	0.2573	0.1908	-0.9550 **	0.7085
*g__Bacillus*	0.1364	0.5816	-0.0776	0.9625**	0.0122	0.9746 **	0.9845**	0.8847 *	0.8892 *	-0.2777	0.4160	0.2858	0.1432	0.0807
*g__Erythrobacter*	-0.2009	-0.8388 *	-0.3895	-0.4988	-0.4574	-0.3803	-0.2490	-0.4772	-0.0985	-0.3110	-0.8858	0.8121 *	-0.2636	0.5843
*g__Euzebya*	0.6410	0.2404	-0.5815	0.7887 *	-0.4978	0.7126	0.9172**	0.9341**	0.5059	-0.7327	0.0789	0.1176	0.6357	-0.3293
*g__Halomonas*	0.4496	0.6023	-0.2307	0.9568 **	-0.1303	0.8892 *	0.9646 **	0.9945 **	0.6669	-0.4160	0.4608	-0.0421	0.4670	-0.3004
*g__Kocuria*	0.7376	-0.6347	-0.7058	-0.5329	-0.7382	-0.6805	-0.3997	-0.1713	-0.8606*	-0.5734	-0.5708	-0.4162	0.7224	-0.6970
*g__Marinicella*	-0.8277*	0.3697	0.9550**	-0.2553	0.9214 **	-0.1596	-0.5091	-0.5568	0.0091	0.9952 **	0.4920	-0.1685	-0.7955 *	0.4185
*g__Marinobacter*	-0.8329 *	0.3395	0.9460**	-0.2843	0.9093 **	-0.1864	-0.5324	-0.5831	-0.0102	0.9917 **	0.4637	-0.1560	-0.8024 *	0.4288
*g__Methylohalomonas*	-0.8229 *	0.3811	0.9575 **	-0.2458	0.9251 **	-0.1517	-0.5020	-0.5471	0.0128	0.9961 **	0.5032	-0.1775	-0.7901 *	0.4104
*g__Mongoliicoccus*	0.3077	0.6794	-0.0977	0.9897 **	0.0028	0.9478**	0.9718 **	0.9674 **	0.7633 *	-0.2937	0.5374	0.0129	0.3275	-0.1808
*g__Nitriliruptor*	0.6682	-0.4769	-0.9327 **	0.1970	-0.9119 **	0.1494	0.4854	0.4511	0.0727	-0.9697 **	-0.6144	0.4026	0.6243	-0.1865
*g__Pelagibius*	-0.7712*	-0.4735	0.2869	-0.5244	0.2097	-0.3365	-0.4555	-0.7318	0.0503	0.3617	-0.4586	0.7293	-0.8093*	0.8922 *
*g__Promicromonospora*	-0.1960	-0.7625 *	-0.4018	-0.3559	-0.4572	-0.2302	-0.0979	-0.3510	0.0486	-0.3540	-0.8372 *	0.8842 *	-0.2591	0.6227
*g__Sphingomonas*	0.7699 *	-0.4555	-0.6073	-0.4429	-0.6267	-0.6157	-0.3682	-0.0882	-0.8546 *	-0.4881	-0.3769	-0.6025	0.7688 *	-0.8226 *

SWC, soil water content (%). VC, vegetation coverage (%). NS, number of *Suaeda glauca* (m^2^). HS, height of *Suaeda glauca* (cm). AGB, above-ground biomass (kg ⋅ m**^–^**^2^). An asterisk (*) on the columns indicates that the difference is significant (*p* < 0.05), and ** indicates that the difference is very significant (*p* < 0.01).

Environmental factors had significant effects on the abundance and diversity of rhizosphere bacterial community. Our results showed that the number of bacterial species in the different treatments was extremely significantly negatively correlated with the SWC and VC and extremely significantly positively correlated with the AGB (Table 3). The richness and evenness indices were extremely significantly negatively correlated with the SO_4_^2–^ concentration and significantly negatively correlated with the SWC, K^+^ and Mg^2+^ concentrations and VC. These correlations indicated that the number and diversity of the bacteria were inhibited by both the SWC and VC in *S. glauca*-dominated communities in the Hetao Irrigation District area.

Dominant populations played a key role in stabilizing the structure and function of bacterial communities, which were mainly affected by surrounding environmental factors. Therefore, the species with the highest coverage of identifiable microorganisms at the phylum level and species with detailed taxonomy and high identifiability at the genus level were selected for correlation analysis with soil and land surface indicators. The results showed that the number of microorganisms in the most common phyla was significantly correlated with the soil EC and HCO_3_^–^+CO_3_^2–^ and Na^+^ concentrations and extremely significantly correlated with the SWC and VC. *Proteobacteria* and *Chloroflexi* exhibited significant positive correlations with the soil EC and Na^+^ and HCO_3_^–^+CO_3_^2–^ concentrations and extremely significant negative correlations with the SWC and VC. *Gemmatimonadetes* was significantly positively correlated with the EC and Na^+^ concentration. *Acidobacteria* showed significant positive correlations with the EC and HCO_3_^–^+CO_3_^2–^ concentration and extremely significant negative correlations with the SWC and VC. These results indicate that the number of microorganisms in these phyla was mainly affected by the soil EC, Na_2_CO_3_, NaHCO_3_, SWC and VC. In addition, *Bacteroidetes* and *Firmicutes* showed an extremely significant correlation with Cl^–^ and significant correlations with the Ca^2+^and Mg^2+^ concentrations, although the latter correlations were negative for *Bacteroidetes* and positive for *Firmicutes*. Only *Actinobacteria* had a significant negative correlation with NS and a significant positive correlation with HS.

The correlation analysis between the bacterial genera and environmental factors showed that the number of microbial genera in the area of *S. glauca* in the Hetao Irrigation District was closely related to the ionic composition of different salts. The most abundant genera were divided into two classes. One class showed an extremely significant or significant correlation with one or multiple factors (soil EC, Na^+^, CO_3_^2–^ and HCO_3_^–^) and included bacterial genera such as *Marinicella, Marinobacter*, *Methylohalomonas* and *Nitriliruptor*. The other class exhibited an extremely significant or significant correlation with one or multiple factors (soil K^+^, Ca^2+^, Mg^2+^ and SO_4_^2+^) and included bacterial genera such as *Bacillus*, *Euzebya*, *Halomonas* and *Mongoliicoccus*. *Kocuria* was the only genera that was significantly positively correlated with Cl^–^, and this genus was not extremely significantly correlated with any other indicators. Two genera, namely, *Erythrobacter* and *Promicromonospora*, were significantly correlated with the pH and HS. Among the aboveground indicators, VC exerted the greatest effect on bacteria genera and showed a higher correlation. Interestingly, the bacteria significantly related to VC exhibited a significant correlation with soil water. The above-described analysis showed that EC, soluble ions (Na^+^, CO_3_^2–^ + HCO_3_^–^, K^+^, Ca^2+^, Mg^2+^, SO_4_^2+^), SWC and VC were the main factors affecting the bacterial community structure in the rhizosphere soil of *S. glauca.*

## 4 Discussion

### 4.1 Differences in rhizosphere bacterial characteristics among the sampling sites

Soil microorganisms were highly important components and the most active constituents in the terrestrial soil system and played an indispensable role in soil formation and development, organic matter transformation, ecosystem balance, soil environmental purification and bioremediation (Gans et al., 2005). The results from the current study showed that although all sampling sites were located in the Hetao irrigation area, and the vegetation was *S. glauca* in all cases, the microbial characteristics exhibited extremely significant differences. Microbial differences affected the soil nutrient cycling process, the decomposition of organic matter and the absorption and utilization of nutrients by plants, as well as soil biodiversity, and then affected the stability and disturbance resistance of soil ecosystem (Hartmann and Six, 2023). Because the present study used select areas of *S. glauca*-dominated vegetation, the soil microbial characteristics under the same plant conditions should be mainly related to the different ecological environments and vegetation growth characteristics.

Soil salt content, pH value and nutrient content were the key environmental factors that affected the structure and function of soil microbial community. Soil nutrients were the main source of energy and nutrients for microorganisms, and changes in soil nutrient content directly affected the growth and metabolic activities of microorganisms (Bardgett and Shine, 1999). High salinity affected the stability of microbial cell membrane and the water balance inside and outside the cell, thus affecting the physiological metabolic process of microorganisms (Zhang et al., 2019). Soil pH affected the availability of nutrients and the activity of enzymes, which in turn affected the absorption of nutrients by microorganisms (Xiang et al., 2023). Different ecological environments directly or indirectly affected the composition and structure of soil microorganisms by affecting soil nutrient content, salt content, pH and other factors.

Our study showed that in severely saline-alkali land, the underground microorganisms of *S. glauca* vegetation were strongly affected by the HS, AGB, pH, HCO_3_^–^+CO_3_^2–^ and Na^+^ concentrations and EC. In moderately saline-alkali land, the underground microorganisms of *S. glauca* vegetation were strongly affected by the K^+^, Ca^2+^, SO_4_^2–^ and Mg^2+^ concentrations, and in slightly saline-alkali land, the underground microorganisms of *S. glauca* vegetation were strongly affected by the soil K^+^, Ca^2+^, SO_4_^2–^, and Mg^2+^ concentrations, VC and SWC. Differences between the sampling sites were subjected to the dual effects of aboveground vegetation characteristics and soil physicochemical properties (Ma et al., 2016; Yang et al., 2019).

### 4.2 Diversity of rhizosphere bacterial communities at different sites

In general, increased salinity lead to soil osmotic stress that limited microbial growth and activity, resulting in declines in the microbial population and biomass (Kamble et al., 2014). However, this study found that the richness and diversity of bacteria did not decrease continuously with an increase in salinity, where the species richness and diversity of rhizosphere bacteria were higher in severely saline-alkali land than in slightly and moderately saline-alkali land, which was similar to the results reported by Zhao J. et al. (2020). On the one hand, high salinity stress may indeed increase the richness and diversity of rhizosphere soil bacterial communities. Some studies have shown that salinity altered the root distribution of halophytes and increased the diversity and richness of inter-rooted soil bacteria (Yang et al., 2016). In addition, a recent study found that root exudates played a major role in influencing the richness and diversity of inter-rhizosphere soil bacteria (De Vries et al., 2020). Root exudates can provide nutrients and carbon sources, thus promoting the growth and reproduction of rhizosphere bacteria. They can also secrete phenols, amino acids and specific secondary metabolites to promote or inhibit the development of specific bacterial communities, which significantly affected the abundance and diversity of rhizosphere bacterial communities (Yang et al., 2020). Another explanation was that *S. glauca* in severely saline-alkali land was subjected to high salt stress, which stimulated its root systems to secrete more chemicals and thus exerted a strong driving force on the growth of the inter-rhizosphere soil bacterial community, greatly increasing the richness and diversity index of the bacterial community (Mimmo et al., 2014; Jia et al., 2017). The abundance and diversity of rhizosphere bacterial communities were increased under high salt stress, which contributed to enhance the salt tolerance of plants and helped plants better adapt to high salt environment and other abiotic stresses. At the same time, it can enhance the circulation and availability of nutrients in the soil, maintain various physiological functions of plants under salt stress, and improve plant growth. In addition, it can also enhance the stability of the rhizosphere ecosystem and its resistance to environmental changes, helping to maintain long-term soil health and productivity.

Some studies have suggested that the SWC was the key factor mediating soil bacterial community (Banerjee et al., 2016). However, the effects of soil water content on the diversity and richness of the rhizosphere bacterial community were controversial (Manzoni et al., 2012). In our study, the RDA indicated that the diversity and richness of the rhizosphere bacterial community were significantly negatively correlated with the SWC and VC, and this finding may have been obtained because an increase in the SWC may reduce the gas diffusion rate and limit the supply of oxygen, which may reduce the niche of bacteria and in turn adversely affect the diversity of rhizosphere microorganisms (Blagodatsky and Smith, 2012). This study saline-alkali land revealed an extremely significant positive correlation between the VC and SWC. VC may reduce the soil surface area available for evaporation and water loss and thereby affect the SWC. The Hetao Irrigation District in Inner Mongolia was located in arid and semi-arid areas, where the rainfall rate was markedly slower than the evaporation rate, and the bare land often aggravated the evaporation of soil moisture and reduced the water holding capacity of soil (Ruan et al., 2020). Therefore, the VC may indirectly affect the richness and diversity of rhizosphere bacteria by regulating the soil water holding capacity. The VC and SWC in the S3 plot were the lowest, and this special condition may promote the coexistence of multiple bacteria, so the S3 plot had a high diversity and abundance of bacterial communities.

In addition, RDA showed that SO_4_^2–^ had a significant effect on the bacterial community diversity, and an extremely significant negative correlation was detected between these variables. Some studies have suggested that SO_4_^2–^ can change soil properties, leading to changes in the soil volume, and then affected the structure and diversity of microbial communities (Baghabra Al-Amoudi et al., 2018). The effect of SO_4_^2–^ on the bacterial community diversity has not yet reached a consensus, and the specific reasons are unclear and need further research.

### 4.3 Main rhizosphere bacterial communities at different sites

Our investigation results showed that a large number of bacteria affiliated with the phyla *Proteobacteria*, *Actinobacteria*, *Gemmatimonadetes*, *Bacteroidetes* and *Chloroflexi* coexist with *S. glauca* vegetation in the saline-alkali land in the Hetao Irrigation District. These phyla were thus considered the main bacterial phyla in the saline-alkali areas of *S. glauca* in the Hetao Irrigation District. Among them, *Proteobacteria* was the most important dominant phylum among all sampled sites, with a high percentage of 39.55% to 52.34%, which was consistent with previous results reported for other saline plants (Shi et al., 2015; Tian and Zhang, 2017). *Proteobacteria* grew rapidly, had strong metabolic diversity, and can therefore survive in some extreme environments (Xu et al., 2022). Many bacteria of *Proteobacteria* can fix nitrogen and produce plant-friendly polycyclic aromatic hydrocarbons (Johnston-Monje et al., 2016; Liu et al., 2020). Some studies have found that a higher nitrogen level within a certain range was more conducive to the growth of *S. glauca* (Song et al., 2020). Therefore, *Proteobacteria* as the dominant bacteria was often found in the rhizosphere of *S. glauca*.

This study found that the richness of *Proteobacteria* was significantly positively correlated with soil salinity, which showed that the S3 plots with the highest salt content had the largest number of *Proteobacteria*. This finding is similar to that reported by Zheng et al. (2017), who found that the relative abundance of *Proteobacteria* was higher under high salinity than that in soil with low and medium salinity. Previous studies demonstrated that some *Proteobacteria* were halotolerant and could cope with high salinity through the uptake and accumulation of compatible solutes (Sand et al., 2014). The above-described studies supported the finding that *Proteobacteria* may be resistant to a high salt environment, which also explained why *Proteobacteria* was the dominant bacteria in highly saline soil.

*Acidobacteria* and *Chloroflexi* were bacterial groups that were widely distributed in ecosystems, and they played important ecological roles in a variety of environments. Some members of *Acidobacteria* interacted with plants and played an important role in the biogeochemical cycle. Studies have shown that there were high frequency of plant growth promoting traits (PGPT) genes in *Acidobacteriaceae*, *Bryobacteraceae*, *Koribacteraceae*, and *Pyrinomonadaceae* Families, and these genes include genes involved in nitrogen fixation, phosphate solubilization and other processes (Gonçalves et al., 2024). *Chloroflexi* were involved in a variety of biogeochemical cycle processes, including carbon, nitrogen and sulfur cycle processes such as CO_2_ fixation, degradation of macromolecules such as cellulose, oxidation of NO_2_^–^, and oxidation of SO_3_^2–^ (Xian et al., 2020).

In addition, *Acidobacteria* and *Chloroflexi* were recognized as oligotrophs and tended to dominate under oligotrophic conditions (Zeng et al., 2016). Fierer et al. (2007) found that *Acidobacteria* and *Chloroflexi* were more abundant in soils with low C availability. Salinity could inhibit soil carbon accumulation (Zhao et al., 2017). Therefore, in the current study, *Acidobacteria* and *Chloroflexi* were significantly enriched in the S3 plots likely due to the influence of high salinity.

Members of the phylum *Actinobacteria* were also commonly found in saline-alkaline soils. Some members acted as decomposers, participating in the decomposition of inter-root soil organic matter and producing active substances that stimulated vegetation growth, such as cytokinin (Shi et al., 2020), and the presence of *Actinobacteria* may enhance the stress resistance of *S. glauca* in saline environment.

*Actinobacteria* was previously described as copiotrophs that thrived in environments with high carbon availability (Mickan et al., 2019). Zhu et al. (2018) found that the relative abundance of *Actinobacteria* was higher in soils with a higher SOC content. In addition, *Actinobacteria* existed in the soil mainly in the form of spores or hyphae, which had strong drought resistance and were more abundant in the soil environment with low water content (Nan et al., 2020). In the present study, the level of *Actinobacteria* in rhizosphere soil was relatively high in the S4 plots, which was closely related to the low water content and the proximity of these plots to grazing land and affected by livestock manure.

*Gemmatimonadetes* has been identified as the common phyla found in the rhizosphere of *S. glauca*. Studies have found that some members of *Gemmatimonadetes* can be capable of anaerobic photosynthesis, which may have an important impact on the carbon cycling in soil. However, many members have not yet been cultured, which limited the in-depth understanding of their ecological functions and metabolic characteristics (Zeng et al., 2014; Zhao Q. et al., 2020). In the current study, a significant positive correlation was found between the pH and relative abundance of *Gemmatimonadetes*, and *Gemmatimonadetes* was significantly enriched in the S1 plots with the highest pH. At present, there were few reports on *Gemmatimonadetes* in rhizosphere soil. The characteristics and optimal environmental conditions for *Gemmatimonadetes* species warranted further investigation.

*Firmicutes*, the dominant bacteria in the rhizosphere of *S. glauca*, was often involved in the decomposition of organic matter and microbial N fixation (Li et al., 2021). The enrichments of these bacteria in rhizosphere soils were of great significance for promoting the growth of *S. glauca* and the release of soil nutrients. It has been reported that *Firmicutes* is greatly affected by the soil pH (Karimi et al., 2018), but the present study revealed that the pH did not significant affect *Firmicutes*. This may be related to the differences in soil types and the dual effects of soil and vegetation factors in the studied area. In some research areas, soil pH may interact with soil physicochemical factors (such as available nutrients, saline-alkali ions, soil organic matter content). These complex interactions may mask the independent effect of pH on *Firmicutes* (Dai et al., 2019). Different plants played a leading role in influencing *Firmicutes* through their rhizosphere processes (such as root exudates, plant residue decomposition), which in turn affected the response of *Firmicutes* to pH (Xie et al., 2022).

As can be seen above, there were differences in the relative abundance of dominant phylum among treatments, which was due to the significant differences in soil characteristics of each treatment, such as soil pH value, salt content, organic matter content, and these differences would directly affect the abundance of rhizosphere bacteria. In addition, the number and growth status of *S. glauca* varied among treatments, which may affect the release amount and chemical properties of root exudates, and may promote or inhibit the growth of different bacteria, thus affecting the relative abundance of bacteria.

Notably, these dominant bacteria mitigated the damage of soil saline-alkali stress on *S. glauca* and improved the adaptability of *S. glauca* to saline-alkali environment by releasing active substances that promoted plant growth and participating in nutrient cycling and transformation processes (Borruso et al., 2014).

Further study was needed to clarify how bacteria mitigated salt stress and which bacteria played a major role in the same plant species in similar ecological environments. In terms of genus classification, many *Halomonas* species were found to be distributed across all the treatments. Many members of this genus exhibited high salt tolerance (Arahal et al., 2002; Zou and Wang, 2010) and had gradually replaced the salt-intolerant bacteria to occupy a dominant position in the rhizosphere during long-term exposure to saline-alkali stress. This finding indicated that *Halomonas* was among the bacterial markers widely distributed in saline-alkali *S. glauca* land in the Hetao Irrigation District. *Halomonas* was related to the function of the nitrogen cycle, and its enrichment in rhizosphere soil can improve the uptake and utilization of nitrogen by plants (Miao et al., 2015). This genus can also secrete exopolysaccharides to protect plant roots from excessive salinity (Upadhyay et al., 2011), which was of great significance to ensure the survival of *S. glauca* under adverse conditions and promote the ability to resist external salt stress. The bacterial genera *Nitriliruptor* and *Pelagibius*, which were found at a relatively high abundance in the different treatments, might also exist as marker microorganisms for areas of *S. glauca*. Many of the sequences denoted “unclassified” at the genus level were unidentified species, but were present in large numbers after the different treatments. Whether special salt-tolerant growth-promoting bacteria occur in areas of *S. glauca* needed to be further verified by isolation, cultivation and inoculation studies.

### 4.4 Main factors driving rhizosphere bacterial community structure

Many studies have shown that both biotic and abiotic factors such as soil physical and chemical properties, nutritional status, water, plant genotype and temperature played an important role in regulating the microbial community structure (Martiny et al., 2011; Ramirez et al., 2012; Bell et al., 2015; French et al., 2016). The results of the current study showed that the number of bacterial species under the different treatments was affected by the SWC, VC and AGB and that the richness and evenness indices were affected by the SO_4_^2–^, SWC, K^+^, Mg^2+^ and VC.

In saline-alkali environments, salinity had significant effects on the activity, diversity and structure of the soil microbial communities (Chandrasekaran et al., 2014; Yang et al., 2016). In general, salt stress can reduce the diversity and activity of soil bacteria in the plant rhizosphere (Ibekwe et al., 2010; Bencherif et al., 2015). However, for salt-tolerant *S. glauca*, our study found that salt stress can increase the diversity of the rhizosphere soil bacteria, and this finding may have been obtained because an increase in the salt content promotes the proliferation of salt-tolerant bacteria or halobacteria (Yang et al., 2016).

It was worth noting that salt ions such as K^+^, Mg^2+^ and Na^+^ exerted different effects on different bacterial communities in the rhizosphere. Different ion compositions drived changes in the bacterial communities in the rhizosphere and ultimately had a significant impact on the structure and diversity of bacterial communities in the rhizosphere.

However, few studies had investigated the effects of different saline-alkali ions. Our current study showed that the number of microorganisms at the genus level in the area of *S. glauca* in the Hetao Irrigation District was most closely related to the ionic composition of different salts and that the most abundant genera were divided into two classes. One class was correlated with one or multiple factors among the soil EC, Na^+^, CO_3_^2–^ and HCO_3_^–^, and the other class was correlated with one or multiple factors among K^+^, Ca^2+^, Mg^2+^ and SO_4_^2+^ in the soil; the correlations were extremely significant or significant. These saline-alkali ions showed a positive correlation with the bacterial community, indicating that the rhizosphere bacterial community had the ability to adapt to or alleviate various saline-alkali stresses. Compared with soil saline-alkali ions, the correlation between total salinity (EC), alkalinity (pH) and soil bacterial communities was weaker. This is similar to the research results of Zhang et al. (2021), soil saline-alkali ion composition was an important driving factor affecting the bacterial community of saline-alkali soil, rather than just being mainly affected by salinity, alkalinity (pH) and other soil nutrients. The exact mechanisms and the way in which saline-alkali ion affected the microbial communities remained unknown and required further investigation.

Plants exhibited active selectivity for the soil bacterial community structure in the rhizosphere (Kowalchuk et al., 2002), and differences in the bacterial compositions and structures were found among the rhizosphere of different plant types (Ziegler et al., 2013). Herein, all vegetation communities were dominated by *S. glauca*; thus, the differences in the rhizosphere bacterial communities were more attributable to differences in the ecological environment and plant growth status. Some studies had determined that the soil bacterial community structure were affected by plants, with both showing a certain specificity for plant species and for the growth and development stage (Li et al., 2014). Plants attracted some specific bacterial populations to be enriched in the rhizosphere through root exudates (such as carbohydrates, amino acids, flavonoids), and the morphology (number, length, diameter) and structure of plant roots provided attachment points for different rhizosphere bacteria. Different types of plant or plants at different growth stages secreted different root exudates, and there were differences in the morphology and structure of plant roots, which had different effects on soil rhizosphere bacterial community structure (Li et al., 2022). The present study revealed that due to differences in the VC and AGB of the same plant species, differences in microorganisms exist. This finding indicated that plant activities have changed the rhizosphere soil microenvironment (Ciccazzo et al., 2014), but the mechanism of action and the extent of the changes still needed to be further analyzed.

## 5 Conclusion

This study found that the soil bacterial communities in the rhizosphere of the *S. glauca-*dominated communities in the Hetao Irrigation District were mainly composed of 16 phyla (*Proteobacteria, Actinobacteria, Bacteroidetes, Gemmatimonadetes, Chloroflexi, Acidobacteria, Firmicutes, Planctomycetes, Deinococcus-Thermus, Verrucomicrobia, Saccharibacteria, Cyanobacteria, Nitrospirae, JL-ETNP-Z39, Parcubacteria, and Chlorobi*). At the genus level, the dominant genera of the bacterial community were *Halomonas*, *Nitriliruptor*, *Euzebya* and *Pelagibius*.

Significant differences in the richness and diversity of bacteria in rhizosphere soil were observed under different treatments, and the richness and diversity of the bacterial communities in severely saline-alkali land were found to be higher than those in slight and moderately saline-alkali land.

In addition, the soil microbial characteristics under the same plant conditions were mainly related to the soil physicochemical properties and vegetation characteristics. The EC, soluble ions (Na^+^, CO_3_^2–^ + HCO_3_^–^, K^+^, Ca^2+^, Mg^2+^, SO_4_^2+^), SWC and VC were identified as the main driving factors affecting the changes in the rhizosphere bacterial community structure.

The rhizosphere dominant bacterial community produced plant growth regulating substances, participated in the cycling process of nutrient elements and the decomposition of organic matter, so it played an important role in promoting the growth of *S. glauca* and helping it adapt to the saline-alkali environment.

## Data availability statement

The datasets presented in this article are not readily available because of the upgrade of the data repository and the excessive memory of the data, and data not being stored properly. Requests to access the datasets should be directed to 18800432218@163.com.

## Author contributions

RD: Conceptualization, Data curation, Formal analysis, Investigation, Validation, Methodology, Software, Writing – original draft. XW: Data curation, Formal analysis, Writing – review and editing. YL: Resources, Supervision, Writing – review and editing. HZ: Investigation, Writing – review and editing. XL: Formal analysis, Supervision, Writing – review and editing. JS: Investigation, Writing – review and editing. FC: Investigation, Software, Writing – review and editing. WF: Investigation, Writing – review and editing. HP: Writing – review and editing. JW: Conceptualization, Data curation, Funding acquisition, Methodology, Project administration, Writing – review and editing.
